# The impact of spatial correlation on methylation entropy with application to mouse brain methylome

**DOI:** 10.1186/s13072-023-00479-6

**Published:** 2023-02-04

**Authors:** Xiaowei Wu, Joung Min Choi

**Affiliations:** 1 Department of Statistics, Virginia Tech, 250 Drillfield Drive, Blacksburg, VA 24061 USA; 2 Department of Computer Science, Virginia Tech, 620 Drillfield Drive, Blacksburg, VA 24061 USA

**Keywords:** Bisulfite sequencing, DNA methylation, Methylation entropy, Methylation probability, Spatial correlation

## Abstract

**Background:**

With the advance of bisulfite sequencing technologies, massive amount of methylation data have been generated, which provide unprecedented opportunities to study the epigenetic mechanism and its relationship to other biological processes. A commonly seen feature of the methylation data is the correlation between nearby CpG sites. Although such a spatial correlation was utilized in several epigenetic studies, its interaction to other characteristics of the methylation data has not been fully investigated.

**Results:**

We filled this research gap from an information theoretic perspective, by exploring the impact of the spatial correlation on the methylation entropy (ME). With the spatial correlation taken into account, we derived the analytical relation between the ME and another key parameter, the methylation probability. By comparing it to the empirical relation between the two corresponding statistics, the observed ME and the mean methylation level, genomic loci under strong epigenetic control can be identified, which may serve as potential markers for cell-type specific methylation. The proposed method was validated by simulation studies, and applied to analyze a published dataset of mouse brain methylome.

**Conclusions:**

Compared to other sophisticated methods developed in literature, the proposed method provides a simple but effective way to detect CpG segments under strong epigenetic control (e.g., with bipolar methylation pattern). Findings from this study shed light on the identification of cell-type specific genes/pathways based on methylation data from a mixed cell population.

## Background

**Fig. 1:**
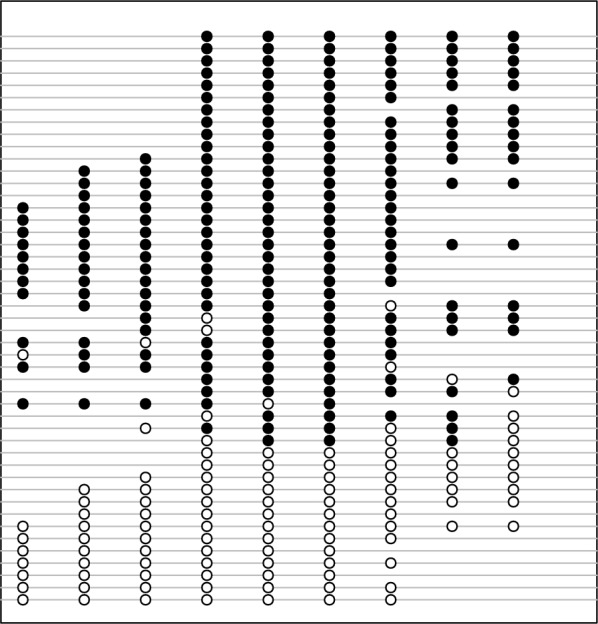
An example of methylation states on a genomic region of nine CpG dinucleotides obtained from promoter region (95 bp) of prodynorphin gene in human brain [4]. Each line represents a sequencing read, with open and filled circles indicating unmethylated and methylated CpG sites, respectively

DNA methylation has been recognized as a key process underlying epigenetics, which involves transferring methyl groups to cytosine bases of the DNA molecule [1]. As the gold-standard for detecting DNA methylation, bisulfite sequencing combines bisulfite treatment with routine sequencing to determine the methylation state at single-nucleotide resolution [2, 3]. By checking the presence of cytosines or thymidines on the mapped short reads for bisulfite-treated DNA, the methylation state of each CpG dinucleotide is obtained. At the read level, the combination of methylation states of neighboring CpG dinucleotides contains important information, which may be used to uncover the role of each genomic locus in the epigenetic control mechanism. As an illustrative example, Fig. 1 shows the methylation states on a CpG segment of nine dinucleotides obtained from the promoter region (95 bp) of the prodynorphin gene in human brain [4]. An immediate observation from this example is that the CpG sites that are spatially close together are likely to be in the same methylation state. Such a phenomenon of *co-methylation* over short (e.g., up to 1–2 kb) distances has been reported in literature [5–7], and utilized to predict [8] or impute [9] DNA methylation, however, its impact on the characteristics of the methylation data has not been fully investigated.

For a single CpG site, its methylation state can be modeled with a Bernoulli random variable whose distribution is featured by the success probability. When it comes to multiple CpG sites (i.e., a CpG segment), appropriate characteristics are needed to summarize the joint distribution of the combinatorial methylation states (not necessarily independent) [10]. One of such characteristics is the methylation entropy, which is a quantitative measure of the variability in DNA methylation shown in sequencing reads [11, 12]. Generally speaking, low entropy indicates less stochasticity, which, in the context of methylation, refers to strong epigenetic control such as allele specific methylation (ASM) [13, 14] or cell-type specific methylation (CSM) [11]. On the other hand, high entropy corresponds to more stochastic methylation events. Therefore, CpG segments with low methylation entropy tend to be biologically more interesting as they may represent differentially methylated regions (DMRs) [15] or serve as potential markers for CSM [16]. Despite the research progress, there is, however a lack of quantitative assessment on how methylation entropy is related to the underlying probability parameter, especially with the involvement of co-methylation. This motivates us to fill such a research gap and look into the resulting application on real data of DNA methylation. It is worth pointing out that, the characteristic for summarizing the distribution of methylation state may refer to either parameter of the probabilistic model or statistic calculated from the methylation data. Though sometimes misused in literature, these two implications need to be distinguished to avoid possible misunderstanding in research. For clarification, we provide statistically rigorous definitions in the "Methods" section for the methylation-related parameters and statistics.

In this paper, we first obtained an explicit relation between the two parameters, the methylation probability and the methylation entropy, under the assumption of independent CpG sites. We then proposed a goodness-of-fit test to check the existence of spatial correlation on the CpG segment under consideration. More generally, in the presence of spatial correlation, we managed to derive the joint distribution of the methylation states on the CpG segment, and based on which attained the relation between the two parameters numerically. Simulation studies validated the proposed test and pinpointed several factors that affect the power of the test. Through additional simulations, we compared the analytical relation between the two parameters with the empirical relation between the two corresponding statistics, to illustrate the impact of spatial correlation. The proposed method was applied to a published dataset of mouse brain methylome to investigate the behavior of the observed methylation entropy as a function of the mean methylation level. Our real data analysis showed that, the proposed method was able to identify CpG segments exhibiting bi-modal methylation patterns, which appeared to be related to DMRs detected between neuron and glia cells. We further examined how the proportion of the identified CpG segments changes from the 2-week stage to the 4-week stage in the mouse front cortex.

## Methods

### Methylation probability, methylation entropy, and their relation in the absence of spatial correlation

The key parameter for describing DNA methylation is the methylation probability (MP) of each CpG site, defined as the probability of observing a methylated cytosine on the site. For a CpG site under consideration, its methylation state can be described by a random variable $X\sim \text {Bern}(p)$ with outcome “1” representing methylation and “0” otherwise, where $p\in [0, 1]$ is the MP. Therefore, for a CpG segment involving *n* sites, the methylation states of the *n* contiguous sites form a binary random vector $\varvec{X}=[X_1, X_2, \ldots , X_n]^T$. This random vector represents the “methylation pattern” exhibited on the *n*-CpG segment. Marginally, $X_i\sim \text {Bern}(p_i),\,p_i\in [0, 1],\,1\le i\le n$, but the joint distribution of $\varvec{X}$ also depends on the correlation among the CpG sites. It has been found that, such a between-site correlation, hereinafter called the spatial correlation, is often nonnegligible [5–7] and plays important roles in the analysis of methylation data, such as detecting epigenome-wide association signals [17], discovering regions associated with exposure [18] or with differential/variabe methylation [19], and hunting epigenomic bumps/peaks [20]. We call $\varvec{X}$ a correlated Bernoulli random vector with parameters $\varvec{p}$ and $\varvec{R}$, where $\varvec{p}=[p_1, p_2, \ldots , p_n]^T$ denotes the mean and

$$\begin{aligned} \varvec{R}=\left( \begin{array}{cccc}1&{}r_{12}&{}\cdots &{}r_{1n}\\ r_{21}&{}1&{}\cdots &{}r_{2n}\\ \vdots &{}\vdots &{}\ddots &{}\vdots \\ r_{n1}&{}r_{n2}&{}\cdots &{}1\end{array}\right) \end{aligned}$$

is the correlation matrix, $r_{ij}\in [-1, 1],\,1\le i\ne j\le n$. Note, the range of the correlation matrix is constrained, see details in Prentice [21] and Chaganty and Joe [22]. There are two special methylation patterns that are of particular interest: (1) *homogeneous methylation*, this refers to the case that $p_i=p,\,1\le i\le n$, i.e., the *n* CpG sites share a common MP; and (2) *exchangeable (or compound symmetric) correlation*, corresponding to $r_{ij}=r$ for $1\le i\ne j\le n$, i.e., the spatial correlation is fixed, not depending on the distance between the CpG sites.

The MP at single-CpG-site level plays a critical role in differentiating samples (e.g., purified cells), however, for analyzing epigenetic heterogeneity in mixed samples (e.g., bulk tissues), the MP has its limitations, and other characteristics at the read level are required. One of such characteristics is the methylation entropy (ME), defined as a measure of the average level of “information” or “uncertainty” inherent to the methylation pattern exhibited across contiguous CpG sites [11, 12]. Different with the MP which only characterizes the methylation state at each single CpG site, the ME summarizes the joint distribution of the methylation states at multiple sites in a CpG segment. By leveraging read-level stochasticity, the ME can be used to evaluate the variation of the methylation pattern on the CpG segment.

Consider an *n*-CpG segment fully covered by *m* sequencing reads. In practice, *n* is usually chosen to be a small integer, e.g., $n=4$. This is to guarantee that enough CpG segments can be extracted from real data and the number of sequencing reads in each CpG segment (i.e., *m*) is sufficiently large. The methylation pattern $\varvec{X}$ is a binary random vector, taking a total of $2^n$ possible values. The distribution of $\varvec{X}$ can be expressed as a function of $p_i$ and $r_{ij},\,1\le i\ne j\le n$. Denote the distribution of $\varvec{X}$ by $\{q_0, q_1, \ldots , q_{2^n-1}\}$ such that:

$$\begin{aligned} q_0= \,& {} P(\varvec{X}=[0, 0, \cdots , 0, 0, 0]^T), \\ q_1=\, & {} P(\varvec{X}=[0, 0, \cdots , 0, 0, 1]^T), \\ q_2=\, & {} P(\varvec{X}=[0, 0, \cdots , 0, 1, 0]^T), \\ q_3=\, & {} P(\varvec{X}=[0, 0, \cdots , 0, 1, 1]^T), \\&\vdots&\\ q_{2^n-1}=\, & {} P(\varvec{X}=[1, 1, \cdots , 1, 1, 1]^T), \end{aligned}$$

the ME of the random vector $\varvec{X}$ is defined straightforwardly from the Shannon entropy [23, 24].

#### Definition 1

The methylation entropy of an *n*-CpG segment is defined by

1 $$\begin{aligned} S=-\sum _{i=0}^{2^n-1}q_i\log (q_i), \end{aligned}$$

where $\{q_0, q_1, \ldots , q_{2^n-1}\}$ form the probability mass function of the methylation states of the *n* contiguous CpG sites.

For convenience, base 2 is often chosen for the logarithm so that the ME can be evaluated in the unit of bits. In the absence of spatial correlation, i.e., assuming independence between the CpG sites, an explicit expression of the ME can be derived in terms of the MP.

#### Corollary 1

If the CpG sites on an n-CpG segment are independent, then the methylation entropy

$$\begin{aligned} S=-\sum _{i=1}^n\left[ (1-p_i)\log _2(1-p_i)+p_i\log _2(p_i)\right] , \end{aligned}$$

where $p_i$ is the methylation probability of the ith CpG site.

#### Proof

Denote the methylation states of the *n* contiguous CpG sites on the segment by a random vector $\varvec{X}$. By the assumption of independence between the CpG sites, the distribution of $\varvec{X}$ can be seen as

2 $$\begin{aligned} q_0=\, & {} P(\varvec{X}=[0, 0, \cdots , 0, 0, 0]^T)=\prod _{i\in N}(1-p_i),\nonumber \\ q_1=\, & {} P(\varvec{X}=[0, 0, \cdots , 0, 0, 1]^T)=\left[ \prod _{i\in N\setminus n}(1-p_i)\right] p_n,\nonumber \\ q_2=\,& {} P(\varvec{X}=[0, 0, \cdots , 0, 1, 0]^T)=\left[ \prod _{i\in N\setminus \{n-1\}}(1-p_i)\right] p_{n-1}, \nonumber \\ q_3=\,& {} P(\varvec{X}=[0, 0, \cdots , 0, 1, 1]^T)=\left[ \prod _{i\in N\setminus \{n-1,n\}}(1-p_i)\right] p_{n-1}p_n,\nonumber \\&\vdots&\nonumber \\ q_{2^n-1}=\,& {} P(\varvec{X}=[1, 1, \cdots , 1, 1, 1]^T)=\prod _{i\in N}p_i, \end{aligned}$$

where $N=\{1, 2, \ldots , n\}$. Plugging in (2) to the ME definition (1), the expression of ME is simplified to $S=-\sum _{i=1}^n\left[ (1-p_i)\log _2(1-p_i)+p_i\log _2(p_i)\right]$. That is, under the assumption of independent CpG sites, *S* is simply the *n*-bit binary entropy function. □

Given independence between the CpG sites, for the special case of homogeneous methylation, i.e., $p_i=p$ for all $1\le i\le n$, Corollary 1 simplifies to

$$\begin{aligned} S=-n\left[ (1-p)\log _2(1-p)+p\log _2(p)\right] . \end{aligned}$$

**Fig. 2:**
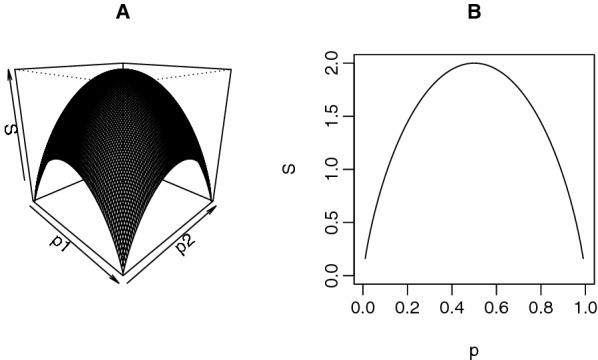
Analytical relation between methylation entropy *S* and methylation probability *p* in a 2-CpG segment under independence assumption. **A** *S* as a function of $p_1$ and $p_2$ on the two CpG sites. **B** Relation between *S* and *p* when $p_1=p_2=:p$

To illustrate the analytical relation in Corollary 1, for a simple example of 2-CpG segment, Fig. 2A shows the ME *S* as a function of the MPs $p_1$ and $p_2$ on the two CpG sites under the independence assumption. In particular, the relation between the ME and the MP for the homogeneous methylation case is shown in Fig. 2B, which is also the curve on the $p_1=p_2$ plane in Fig. 2A.

### Testing for spatial correlation

Based on the distribution of the methylation pattern on a CpG segment, we can check the existence of spatial correlation by a Pearson’s $\chi ^2$ goodness-of-fit test. Under the null hypothesis of no spatial correlation, i.e., $H_0: r_{ij}=0,\,\forall 1\le i\ne j\le n$ or the correlation matrix $\varvec{R}$ is an identity matrix, the distribution of the methylation pattern is provided in (2). Suppose that a total of *m* sequencing reads are collected in bisulfite sequencing to cover this segment, and denote the number of reads showing different methylation patterns corresponding to $\{q_0, q_1, \ldots , q_{2^n-1}\}$ by $O_0, O_1, \ldots , O_{2^n-1}$. These observed counts $O_i$ together with their expected values $E_i,\,0\le i\le 2^n-1$ under $H_0$ form the following contingency table:

**Table:** 

Expected $E_i$	$E_0=mq_0$	$E_1=mq_1$	$\cdots$	$E_{2^n-1}=mq_{2^n-1}$
Observed $O_i$	$O_0$	$O_1$	$\cdots$	$O_{2^n-1}$

The chi-square test can be conducted for goodness-of-fit based on a test statistic

$$\begin{aligned} \chi ^2=\sum _{i=0}^{2^n-1} \frac{(O_i-E_i)^2}{E_i}, \end{aligned}$$

with a degree of freedom $2^n-1-n$ (when $0<p_i<1,\,\forall 1\le i\le n$, a total of *n* MP’s need to be estimated, resulting in a loss of *n* degrees of freedom).

We performed a simulation study (Simulation 1) to evaluate this chi-square test of spatial correlation in terms of empirical type-I error and power. Details of the simulation procedure, evaluation results, and interpretations are provided in the "Results" section.

### Relation between methylation entropy and methylation probability with spatial correlation taken into account

**Fig. 3:**
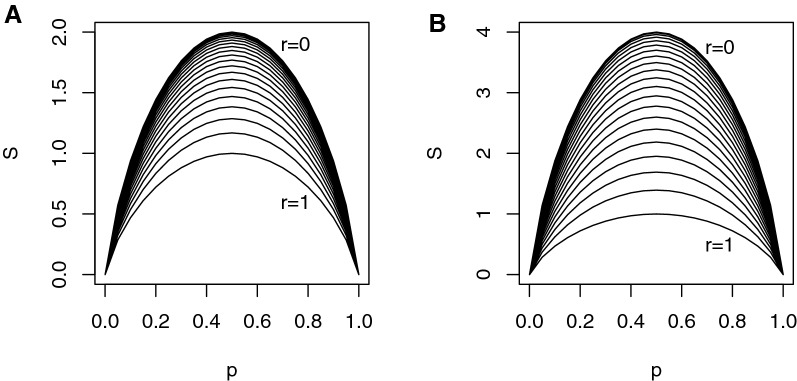
Analytical relation between methylation entropy *S* and methylation probability *p* in an *n*-CpG segment, under different settings of spatial correlation parameter *r*. The 21 curves from top to bottom correspond to $r=0,\,0.05,\,\ldots ,\,0.95,\,1$. For each *r* setting, *S* is shown as a function of *p*. A: $n=2$, under homogeneous methylation (i.e., $p_1=p_2=:p$). B: $n=4$ under exchangeable correlation (i.e., $r_{ij}=r,1\le i\ne j\le 4$) and homogeneous methylation (i.e., $p_i=p,1\le i\le 4$)

In the presence of spatial correlation, the ME is a function of both the MP and the spatial correlation, and intuitively, one would expect that for fixed MP, the ME behaves as a decreasing function of the spatial correlation because larger correlation reduces the stochasticity inherent to the methylation pattern. Such a conjecture can be confirmed by calculating the distribution of the methylation pattern numerically, given the parameters $\varvec{p}$ and $\varvec{R}$ of the correlated Bernoulli distribution. For the convenience of demonstration, here we show the relation of the ME with $\varvec{p}$ and $\varvec{R}$ for the following two cases of homogeneous methylation:

1. ME vs. MP in a 2-CpG segment. Denote the methylation states of the two CpG sites by random variables $X_1$ and $X_2$, respectively. Assuming homogeneous methylation, both $X_1$ and $X_2$ follow the same distribution of $\text {Bern}(p)$. Let $cor(X_1, X_2)=r$, the joint distribution of $(X_1, X_2)$ can be obtained explicitly as:
  $$\begin{aligned} q_0=\,& {} P(X_1=0, X_2=0)=rp(1-p)+(1-p)^2, \\ q_1=\,& {} P(X_1=0, X_2=1)=p(1-p)(1-r), \\ q_2=\,& {} P(X_1=1, X_2=0)=p(1-p)(1-r), \\ q_3=\,& {} P(X_1=1, X_2=1)=rp(1-p)+p^2, \end{aligned}$$
  and consequently, by Definition (1) the ME *S* can be written as a function of *p* and *r*. Figure 3A shows this analytical relation between *S* and *p* for a 2-CpG segment.
2. ME vs. MP in an *n*-CpG segment, $n>2$. When $n>2$, the distribution of the methylation pattern cannot be directly derived from the pair-wise correlations, yet the thresholding method in Emrich and Piedmonte [25] can be adopted to bypass this difficulty. The basic idea is to relate the correlated Bernoulli random vector to a latent multivariate normal (MVN) random vector by dichotomization in each dimension, determine the truncation parameters and covariance matrix of the MVN distribution by using the given parameters of the correlated Bernoulli, and calculate the distribution of the methylation pattern from the joint cumulative distribution function (CDF) of the MVN. For the convenience of demonstration, Fig. 3B shows for a 4-CpG segment the analytical relation among ME, MP, and spatial correlation, under exchangeable correlation and homogeneous methylation assumptions.

The analytical relation between the parameters can be further validated by its analogous counterpart in terms of statistics. For this purpose, we performed a simulation study (Simulation 2) to illustrate the *empirical* relation between two statistics, the *observed* ME (OME) and the mean methylation level (MML), in the simulated data. Denote the simulated methylation data at each CpG segment by an $m\times n$ binary matrix $\varvec{x}$, that is, assuming a total of *m* sequencing reads are collected to fully cover the *n*-CpG segment. The OME statistic $\hat{S}$ is defined as:

$$\begin{aligned} \hat{S}=-\sum _{i=0}^{2^n-1}\frac{O_i}{m}\log \left( \frac{O_i}{m}\right) . \end{aligned}$$

The CpG-site-based ML (i.e., the fraction of methylated cytosines on each CpG site) is the sample mean statistic

$$\begin{aligned} \bar{x}_j=\frac{1}{m}\sum _{i=1}^m x_{ij}, \quad 1\le j\le n, \end{aligned}$$

and the MML statistic is simply the overall sample mean across the *n*-CpG sites. From elementary statistics, the OME and ML are the unbiased estimators of the parameters ME and MP, respectively (MML is also the unbiased estimator of the homogeneous MP). Details of the simulation procedure, results, and interpretations are provided in the "Results" section.

### Real data application

Guided by the theoretical results presented in previous sections as well as the two simulation studies, we applied the proposed method to analyze a published dataset of mouse brain methylome [26]. This dataset includes MethylC-Seq data on mouse frontal cortex during early postnatal, juvenile, adolescent, and adult stages. Our analysis contains three steps: (1) for a given sample, extract all 4-CpG segments under a prespecified depth, (2) calculate the OME and MML statistics for each segment, and draw a scatter plot to show the relation of OME vs. MML, and (3) mark CpG segments which exhibit low OME, non-extreme MML, and strong spatial correlation. As elucidated, the low OME and strong spatial correlation in these segments suggest “bi-modality” in the distribution of methylation pattern, which indicates strong epigenetic control mechanism and can be a potential signal of CSM arising from a mixture of two cell types [16]. We will call such bi-modal distributed CpG segments the *bipolar methylated loci*.

There are two specific aims, association validation and dynamics demonstration, for this real data application. The first one is to check whether the identified bipolar methylated loci are associated with DMRs. For this purpose, we choose two samples that contain bisulfite sequencing data enriched with neuron and glia cells, respectively. The sequencing reads from these two samples are pooled together to form data of a mixture of two cell types, and bipolar methylated loci are then identified from this mixed cell population. Meanwhile, by comparing the mean methylation levels of the CpG segments obtained from the two samples separately, DMRs are detected between neuron and glia cells. We then check the association (or overlap) between the identified bipolar methylated loci and DMRs to validate whether the proposed method helps discover CSM. Second, for dynamics demonstration, we would like to see how the bipolar methylated loci change over time. This is done by choosing two samples from different stages (e.g., 2-week and 4-week) of the mouse frontal cortex data, and comparing the corresponding bipolar methylated loci identified by Steps (1)–(4) above. This comparison sheds light on the dynamics of bipolar methylated loci during mouse brain development. Details of the real data analysis procedure, results, and discussions are provided in the "Results" section.

## Results

### Simulation 1 to evaluate the chi-square test for spatial correlation

Simulation 1 was conducted on a hypothetical *n*-CpG segment with 15 combinatorial settings on the number of CpG sites $n=2, 3, 4$ and the number of sequencing reads $m=20, 40, 60, 80, 100$. Under the null hypothesis, the depth-*m* methylation data $\varvec{x}_j,\,1\le j\le n$ on the *j*th CpG site were generated by sampling from Bernoulli distribution, independently of the other CpG sites. To avoid severe bias in the observed methylation pattern distribution $\{O_i,\,0\le i\le 2^n-1\}$ due to too low/high MP, the success probability of the Bernoulli was limited to random samples drawn from Unif(0.3, 0.7). The empirical type-I error results at nominal level 0.05 are reported in Table 1, based on 10,000 replicates. It can be seen that, for almost all settings of *m* and *n*, the empirical type-I error is not significantly different from the nominal, indicating that the proposed chi-square test is correctly calibrated.

**Table 1:** Empirical type-I error of chi-square test for spatial correlation, at level 0.05

	$m=20$	$m=40$	$m=60$	$m=80$	$m=100$
$n=2$	0.0489	0.0565	0.0505	0.0508	0.0503
$n=3$	0.0459	0.0467	0.0497	0.0475	0.0504
$n=4$	0.0455	0.0472	0.0502	0.0472	0.0520

The empirical type-I error is calculated based on 10,000 simulated replicates under the null hypothesis. The large sample 95% CI for the type-I error at level 0.05 is [0.0457, 0.0543]

The simulation under the alternative involved generating samples from a correlated Bernoulli random vector, which was accomplished by using the thresholding method [25]. Due to the constraint in the parameters of the correlated Bernoulli distribution [21, 22], we limited the spatial correlation to two commonly used structures that are compatible with nonhomogeneous methylation: (i) exchangeable, i.e., $r_{ij}=r$, and (ii) first order autoregressive, or AR(1), i.e., $r_{ij}=r^{|i-j|}$, for $1\le i\ne j\le n$. The basic correlation parameter *r* was set to two different values 0.3 and 0.4. All other parameters used the same setting as in the simulation under the null hypothesis. This simulation was repeated 1000 times, and the empirical power results are reported in Table 2. From this table we can see several clear patterns, showing how the empirical power is affected by each single parameter when all other parameters are fixed:

(i) The empirical power increases with the number of sequencing reads *m*. This is an obvious result because *m* essentially stands for the sample size.
(ii) The empirical power increases with the number of CpG sites *n*. In other words, spatial correlation, if exists, tends to be detected more easily in longer CpG segments than in shorter CpG segments. Intuitively, this is expected since the correlation effect accumulates along the sites of the CpG segment.
(iii) Larger *r* results in higher empirical power, as the basic correlation parameter *r* controls directly the magnitude of the effect.
(iv) Exchangeable correlation yields higher power than AR(1) correlation. This is a simple consequence of (iii).

**Table 2:** Empirical power of chi-square test for spatial correlation, at level 0.05

			$m=20$	$m=40$	$m=60$	$m=80$	$m=100$
EX^a^		$n=2$	0.254	0.497	0.671	0.774	0.847
	$r=0.3$	$n=3$	0.389	0.706	0.885	0.952	0.993
		$n=4$	0.492	0.810	0.950	0.991	0.998
		$n=2$	0.403	0.758	0.898	0.969	0.984
	$r=0.4$	$n=3$	0.670	0.937	0.996	0.997	1.000
		$n=4$	0.772	0.973	0.999	1.000	1.000
AR(1)^a^		$n=2$	0.254	0.497	0.671	0.774	0.847
	$r=0.3$	$n=3$	0.274	0.524	0.773	0.881	0.958
		$n=4$	0.300	0.565	0.761	0.915	0.963
		$n=2$	0.403	0.758	0.898	0.969	0.984
	$r=0.4$	$n=3$	0.505	0.849	0.973	0.994	1.000
		$n=4$	0.535	0.873	0.986	1.000	0.999

The empirical power is calculated based on 1000 simulated replicates under the alternative hypothesis

^a^ *EX* exchangeable,* AR(1)* first order autoregressive

### Simulation 2 to assess the relation between observed methylation entropy and mean methylation level

**Fig. 4:**
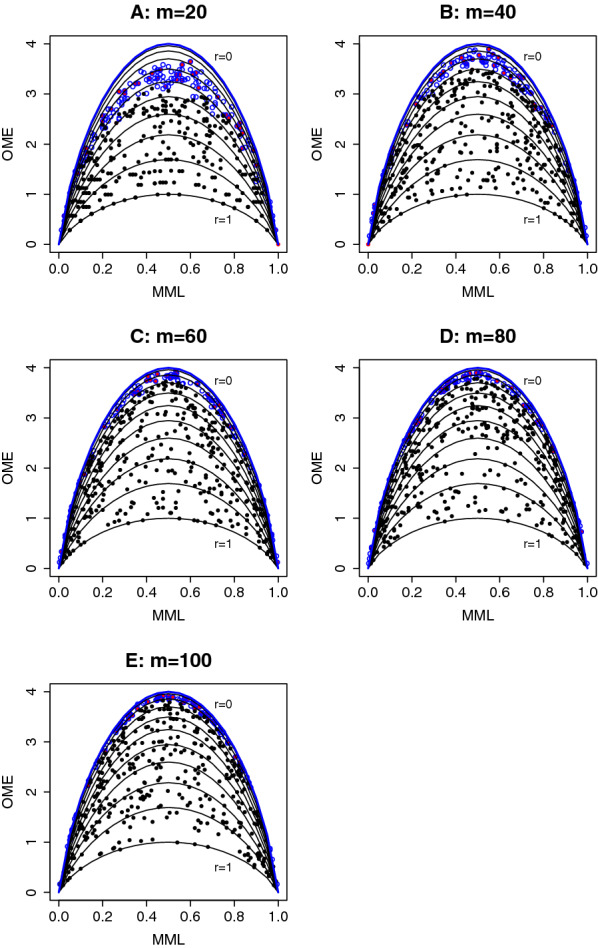
Simulation 2: empirical relation of observed methylation entropy (OME) vs. mean methylation level (MML) in 500 simulated 4-CpG segments, under different sequencing depth *m*. Each dot/circle represents a CpG segment. Red color highlights the 50 CpG segments with no spatial correlation. Blue circles and black dots indicate the absence and presence of spatial correlation in the CpG segment by using the proposed chi-square test. For comparison, the analytical relation between methylation entropy and methylation probability is shown in solid lines, with blue curve standing for no spatial correlation ($r=0$) and 10 black curves from top to bottom corresponding to spatial correlations from weak to strong ($r=0.1,\,0.2,\,\ldots ,\,1$). **A** $m=20$. **B** $m=40$. **C** $m=60$. **D** $m=80$. **E** $m=100$

The purpose of this simulation is to demonstrate the empirical relation between the OME and the MML, as an analogy to the analytical relation between the ME and the MP. The simulation involved a total of 500 hypothetical 4-CpG segments, on which we generated sequencing reads at different sequencing depth $m=20, 40, 60, 80$, and 100. It is worth noting that, since we focus on 4-CpG segments in the simulation (also in real data application), it is not appropriate to consider sequencing depth lower than $2^4$ because the insufficient sample size will result in large deviation of the OME and MML from the corresponding parameters ME and MP. For the convenience of demonstration, the simulation was tailoring to homogeneous methylation and exchangeable spatial correlation with *p* and *r* both drawn from Unif(0, 1) for each CpG segment. All other parameters used the same setting as in Simulation 1. Among the 500 CpG segments, we assumed that 10% had no spatial correlation, and the rest exhibited exchangeable correlation. The OME and MML were calculated for each segment and their relation at different sequencing depth is shown by the scatter plots in Fig. 4. In these scatter plots, each dot/circle represents a 4-CpG segment. The 50 CpG segments with no spatial correlation are highlighted with red color, and the blue circles and black dots indicate whether spatial correlation is detected in the CpG segment by the proposed chi-square test (blue: *p*-value $\ge 0.05$; black: *p*-value $< 0.05$). We see that, for all *m* values in $\{20,40,60,80,100\}$, the 50 CpG segments with no spatial correlation are successfully detected by the test, showing that the type-I error is well controlled. For comparison, the analytical relation between the ME and the MP is overlaid to each scatter plot as solid lines. The blue line at the top corresponds to $r=0$, i.e., no spatial correlation exists, and the 10 black lines are arranged from top to bottom according to $r=0.1, 0.2, \ldots , 1$. It can be seen that, as the sequencing depth increases from 20 to 100, the empirical relation of OME vs. MML approaches closer and closer to the analytical relation of ME vs. MP, and the chi-square test gains more power in detecting CpG segments with spatial correlation (the number of significant* p*-values at $m=20, 40, 60, 80, 100$ are 306, 358, 373, 391, and 383, in contrast to the truth 450). In addition, when the sequencing depth is low, e.g., $m=10$ or 20, due to limited sample size, both the OME and the MML could deviate from the ME and the MP. The effect of small sample size is mainly revealed in the underestimated OME as some methylation patterns (among the total 16) cannot be observed with low sequencing depth. This can be observed clearly from Fig. 4A (for $m=20$) and Fig. 4B (for $m=40$). The OME vs. MML relation on the CpG segments which were tested to have no spatial correlation (blue circles) deviates downwards from the theoretical ME vs. MP relation (blue line), showing underestimated OME in low sequencing depth.

**Fig. 5:**
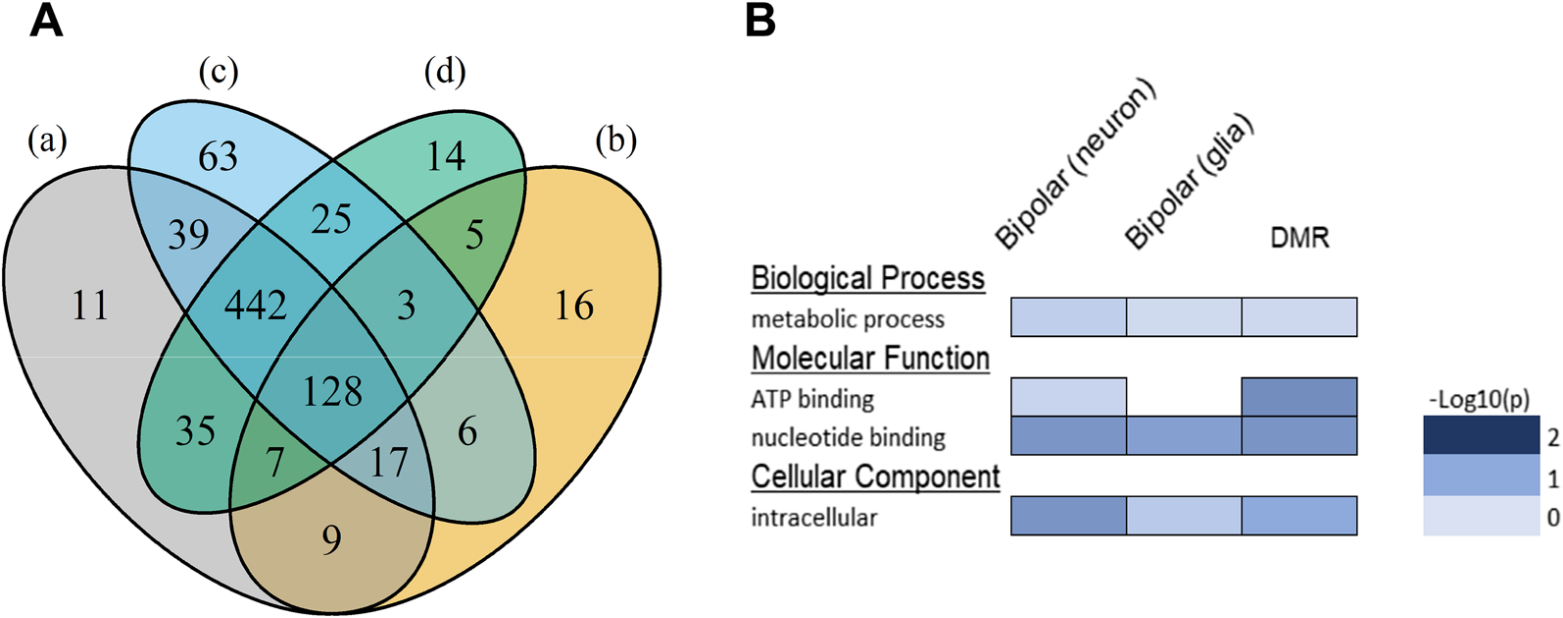
Real data application: validation of association between bipolar methylated loci and differentially methylated regions (DMRs) in neuron and glia samples in mouse brain methylome. **A** Venn diagram showing the overlap among **a** bipolar methylated loci and **b** DMRs, together with **c** bipolar methylated loci identified by using neuron samples and **d** bipolar methylated loci identified by using glia samples. **B** Gene ontology (GO) enrichment analysis of the genes containing bipolar methylated loci and DMRs. *p*-values for GO enrichment were adjusted by Bonferroni correction

### Application to mouse brain methylome

Mouse brain methylome data were downloaded from the NCBI Gene Expression Omnibus (GEO). For the first specific aim, association validation, we used the proposed method to analyze MethylC-Seq data of two samples, SRR921832: NeuN$+$ (neuron) and SRR921839: NeuN− (glia), from 6-week female mouse frontal cortex. These data were generated from populations of nuclei obtained by fluorescence-activated cell sorting. A total of 1012 and 976 4-CpG segments with at least $20\times$ sequencing depth were extracted from the neuron and glia samples, respectively, among which there were 966 in common. Based on pooled sequencing reads of the two samples on the common CpG segments, we obtained 688 bipolar methylated loci under condition “MML $\in (0.2, 0.8)$” and “OME < the theoretical value under spatial correlation $r=0.6$”. On the other hand, among the 966 common CpG segments, 191 were detected to be DMRs by comparing the MML of the two samples via a standard chi-square test. The association between the bipolar methylated loci and the DMRs was evaluated by a Fisher’s exact test based on the following $2\times 2$ contingency table:

**Table:** 

	Bipolar	Non-bipolar	Total
DMR	161	30	191
Non-DMR	527	248	775
Total	688	278	966

The *p*-value for this association test is $4.578\times 10^{-6}$, indicating the existence of strong association, in other words, the bipolar methylated loci are likely (with odds ratio: 2.52) to be DMRs. In addition, the overlap among the bipolar methylated loci and the DMRs, together with the bipolar methylated loci identified by using the neuron and glia samples separately is shown in a Venn diagram in Fig. 5A. Out of the 191 DMRs, 161 (84.3%) were identified as bipolar methylated loci. We note that, the OME threshold $r=0.6$ can be adjusted high or low to allow more stringent or lenient constraint for identifying bipolar methylated loci. For example, if we set $r=0.7$, a total of 478 bipolar methylated loci were detected among which 119 were overlapped with DMRs (*p*-value for association: $9.875\times 10^{-5}$), whereas setting a lower threshold $r=0.55$ resulted in 705 bipolar methylated loci with 166 overlapped with DMRs (*p*-value for association: $4.464\times 10^{-7}$). Figure 5B shows the gene ontology (GO) enrichment analysis result by using DAVID bioinformatics resources tool [27]. Functional relevance was checked for both genes containing bipolar methylated loci in neuron and glia samples, and genes containing DMRs. Gene structure annotations were retrieved from Ensembl genome browser, and gene region was defined to be from transcription start site to transcription end site.

**Fig. 6:**
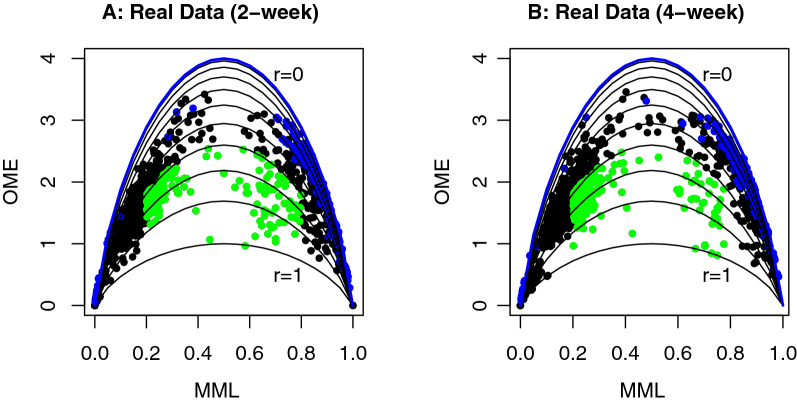
Real data application: demonstration of dynamics of bipolar methylated loci from 2-week to 4-week stages in mouse brain methylome. Each dot represents the empirical relation of methylation entropy (OME) vs. mean methylation level (MML) in a CpG segment with at least 20× sequencing depth. Green dots show the identified bipolar methylated loci. Blue and black dots indicate respectively the absence and presence of spatial correlation decided by the proposed chi-square test. For comparison, the analytical relation between methylation entropy and methylation probability is shown in solid lines, with blue curve standing for no spatial correlation ($r=0$) and 10 black curves from top to bottom corresponding to spatial correlations from weak to strong ($r=0.1,\,0.2,\,\ldots ,\,1$). **A** OME vs. MML in the 2-week sample. **B** OME vs. MML in the 4-week sample

Next, to demonstrate the dynamics of the identified bipolar methylated loci during two stages of mouse brain development, we applied the proposed method on MethylC-Seq data of two samples, SRR921694 in 2-week and SRR921776 in 4-week, from mouse frontal cortex. A total of 1254 and 1200 4-CpG segments with at least 20× sequencing depth were extracted from the two samples, respectively. The MML and OME statistics were then calculated for these CpG segment, and shown in Fig. 6 for the two stages. By using condition “MML $\in (0.2, 0.8)$” and “OME < the theoretical value under strong spatial correlation $r=0.7$”, we identified 186 and 242 CpG segments as bipolar methylated loci (shown as green dots in Fig. 6) in the 2 and 4-week samples, respectively. The blue and black dots in Fig. 6 indicate the presence and absence of spatial correlation decided by the proposed chi-square test. For comparison, the analytical relation between the ME and the MP was added to the scatter plots as solid lines. Comparison between the green dots in Fig. 6A with those in Fig. 6B helps reveal the dynamics of bipolar methylated loci from 2 to 4-week stages in mouse front cortex. To answer the question “is there a significant change in the proportion of bipolar methylated loci from the 2-week to 4-week mouse front cortex” or “is bipolar methylation independent of the two development stages, 2-week and 4-week, of the mouse front cortex”, a standard homogeneity/independence test can be conducted based on the $2\times 2$ contingency table calculated from the two samples:

**Table:** 

	Bipolar	Non-bipolar	Total
2-week	186	1068	1254
4-week	242	958	1200

The corresponding Fisher’s exact test *p*-value (two-sided) is $5\times 10^{-4}$, showing that bipolar methylation does depend on the two development stages of the mouse front cortex. We therefore conclude that, the proportion of bipolar methylated loci in the 2-week mouse front cortex is significantly lower (with odds ratio: 0.69) than that in the 4-week mouse front cortex. In other words, from the 2-week to the 4-week development stage, the mouse front cortex exhibits a decrease of stochasticity in methylation pattern, which confirms previous findings in literature [28].

## Discussion

In this study, we illuminated the analytical relation of methylation entropy vs. methylation probability in a segment of independent or spatially correlated CpG sites. We proposed a chi-square test to check the existence of spatial correlation, and explored how spatial correlation, if exists, impacts with the relation of ME vs. MP. Simulation studies were performed to evaluate the type-I error and power of the proposed test, and assess the empirical relation between the two statistics, the OME and the MML, in companion with the relation between their theoretical counterparts. By analyzing real data from the mouse brain methylome, we identified CpG segments exhibiting bipolar methylation which were shown to be associated with DMRs detected between neuron and glia cells. We also examined the dynamics of bipolar methylated loci in two developmental stages of the mouse front cortex. Compared to other sophisticated methods developed in literature [13, 14, 16], the proposed method provides a simple but effective way to detect CpG segments under strong epigenetic control (i.e., with bipolar methylation). Findings from this study shed light on the identification of cell-type specific genes/pathways based on methylation data from a mixed cell population.

One possible extension of the proposed method for testing spatial correlation is to detect CpG segments with specific correlation structure. For example, instead of testing whether spatial correlation exists in each CpG segment, one may be interested in finding out which CpG segments have exchangeable correlation with $r=0.7$. In this case, the chi-square test can be easily adjusted by updating the null hypothesis, that is, by replacing the joint distribution expression (2) under no spatial correlation assumption with a version under the exchangeable correlation assumption. The updated expression of the joint distribution of methylation pattern can be obtained numerically by using the thresholding method.

Our derivation of the ME expression as a function of the MP under spatial correlation borrows strength from the thresholding method in Emrich and Piedmonte. As a gold-standard approach, this method is flexible to allow arbitrary correlation structure, however, using this method to calculate the joint distribution of methylation pattern involves the solution of non-linear equations via numerical integration, which appears to be computationally inefficient. Other methods may be used as alternatives for calculating the joint distribution or generating random samples of a correlated Bernoulli random vector. For example, Park et al. dichotomizes partial sums of independent Poisson variables [29], Qaqish employs a conditional linear family of distributions [30], Yang and Chaganty adopts Markov chains and multivariate probit models [31], Haynes et al. and Shults develop multinomial sampling [32, 33], Jiang et al. attains three commonly studied correlation structures by combining Bernoulli random variables via computationally efficient algorithms [34].

Another important issue is the choice of *n*, i.e., the number of sites in the CpG segments. In practice, we usually set $n=4$. One may adopt a larger *n* for data with longer sequencing reads. However, there are at least two drawbacks for choosing a large *n*: (1) it will filter out a large number of CpG segments which do not contain sequencing reads with that many contiguous CpG sites, and (2) it will result in a remarkable decrease in *m*, that is, with more CpG sites required in the sequencing reads, fewer sequencing reads will be available in each CpG segment. As an example, in our real data analysis based on sample SRR921694 under depth $20\times$, for $n=4$, the number of CpG segments is 1254 and the average number of sequencing reads of the CpG segments $\bar{m}=59.62$, whereas for $n=6$, the number of CpG segments decreases to 681 and $\bar{m}=56.99$, and for $n=8$, the number of CpG segments further drops to 464 and $\bar{m}=47.17$. Moreover, for large *n*, the calculation of OME will deviate away from its theoretical value ME because some methylation patterns cannot be observed due to limited number of sequencing reads *m* but exponentially increased number (i.e., $2^n$) of possible methylation patterns. In general, given the sample size *m* is sufficiently large, i.e., enough sequencing depth is guaranteed for the CpG segment under consideration, the unbiased statistics OME and MML are able to estimate the ME and MP as accurately as possible. Hence the relation between the OME and MML serves as a good approximation to that between the ME and MP. With limited sequencing depth in real data, the empirical relation of OME vs. MML could be seriously distorted by the nonnegligible estimation error. The investigation of such an issue may be used to guide the quality control procedure in bisulfite sequencing, i.e., to select CpG segments covered by enough sequencing depth, which is worth further exploration in future work.

## Data Availability

All data analyzed in this study are publicly available in NCBI Gene Expression Omnibus (GEO) Accession: GSE47966 [26].

## Abbreviations

MP: Methylation probability
ME: Methylation entropy
ASM: Allele specific methylation
DMR: Differentially methylated region
CSM: Cell-type specific methylation
MVN: Multivariate normal
CDF: Cumulative distribution function
OME: Observed methylation entropy
MML: Mean methylation level
